# Exercise Dose Effects on Body Fat 12 Months after an Exercise Intervention: Follow-up from a Randomized Controlled Trial

**DOI:** 10.1155/2019/3916416

**Published:** 2019-01-20

**Authors:** Christine M. Friedenreich, Yibing Ruan, Aalo Duha, Kerry S. Courneya

**Affiliations:** ^1^Department of Cancer Epidemiology and Prevention Research, CancerControl Alberta, Alberta Health Services, Calgary, Alberta, Canada; ^2^Departments of Oncology and Community Health Sciences, Cumming School of Medicine, University of Calgary, Calgary, Alberta, Canada; ^3^Cross Cancer Institute, CancerControl Alberta, Alberta Health Services, Edmonton, Alberta, Canada; ^4^Faculty of Physical Education and Recreation, University of Alberta, Edmonton, Alberta, Canada

## Abstract

**Background:**

Exercise interventions can result in weight loss, which is associated with reductions in disease risk. It is unknown how the volume of exercise prescribed in a one-time exercise intervention impacts long-term body fatness. We compared 24-month body fat changes among postmenopausal women previously prescribed 300 versus 150 minutes/week of exercise in a year-long exercise intervention trial.

**Methods:**

The Breast Cancer and Exercise Trial in Alberta (BETA) was a two-centred randomized controlled trial in Alberta, Canada. The trial consisted of a 12-month intervention and 12-month observation period. For the intervention, participants were randomized to either a moderate-volume exercise group (150 min/week) or a high-volume exercise group (300 min/week). Participants in this study were 334 inactive postmenopausal women who had been followed-up to 24 months. The primary outcome for this study was 24-month change in total body fat using dual energy X-ray absorptiometry scans. Other measures included weight, waist and hip circumferences, subcutaneous and intra-abdominal fat from computed tomography scans, and lean mass. Researchers were blinded to randomization group when measuring body fat.

**Results:**

Both groups self-reported ∼180 minutes/week moderate–vigorous activity at 24 months. No statistically significant difference was found in total body fat at 24 months between the two groups. Statistically significant effects (comparing high versus moderate groups) were found for BMI (least-square mean change (95% CI): −0.66 (−0.97, −0.36) versus −0.25 (−0.55, 0.05) kg/m^2^, *P*=0.04), waist-to-hip ratio (−0.033 (−0.040, −0.026) versus −0.023 (−0.030, −0.016), *P*=0.05), and subcutaneous abdominal fat area (−32.18 (−39.30, −25.06) versus −22.20 (−29.34, −15.05) cm^2^, *P*=0.04).

**Conclusion:**

Prescribing 300 versus 150 minutes/week of exercise to inactive postmenopausal women resulted in some long-term greater decreases in measures of body composition but no overall differences in total body fat loss. This trail is registered with NCT01435005.

## 1. Introduction

In epidemiologic studies, body mass index (BMI) is associated with significantly higher risks of mortality [1], cardiovascular disease [2], and some types of cancer [3]. In postmenopausal women, higher BMI is associated with an increased risk of invasive breast cancer [4] in part because, after menopause, body fat becomes the primary source of endogenous estrogens and other biomarkers of breast cancer risk [5]. Furthermore, the menopausal transition increases intra-abdominal fat [6], a distinct risk factor for metabolic disease, cardiovascular disease [7], and possibly breast cancer [8].

It is well known that individuals who lose body weight are susceptible to weight regain [9]. Although weight change is often attributed to diet, exercise alone can also induce significant weight loss [10, 11] and exercise is consistently related to better weight maintenance [12]. Observational studies show that postmenopausal women who continue to be physically active are more likely to maintain or lose weight over the long term [13–15]. Conversely, in observational studies, women who are less active during [16] or after [17] weight loss are more likely to regain weight.

Besides weight loss, there are other advantages to exercising in postmenopausal women. Exercise can increase physical fitness [18], improve glucose tolerance [19] and insulin sensitivity [20], decrease intra-abdominal fat [10, 11], and preserve lean mass [10]. The amount of exercise needed to maintain body weight, however, is relatively high. At least 200 minutes/week is recommended for weight maintenance after weight loss [12] and in formerly obese individuals, as much as 60–90 minutes/day moderate activity or 35 minutes/day vigorous activity most days of the week may be necessary [21]. Overall, these durations exceed 150 minutes/week, the minimum recommendation for chronic disease prevention [22].

One clinical strategy for maximizing weight and body fat loss may be to prescribe a high amount of exercise upon initiating an exercise program. An exercise routine >150 minutes/week instilled at the time of initiating an exercise program may be habit-forming, resulting in lower body fat over the long term. We previously reported significantly greater reductions in total body fat, subcutaneous abdominal fat, and waist-to-hip ratio in postmenopausal women randomized to a high versus moderate dose of aerobic exercise for 12 months (300 versus 150 minutes/week moderate–vigorous intensity) [23]. In this paper, we report whether or not these effects were maintained 12 months after the intervention. We hypothesized that at 24 months, body fat change from baseline would still be significantly greater for the high versus moderate group.

Unlike one previous study of this kind [24], we obtained valid measures of whole body and abdominal fat using computerized tomography (CT) and dual-energy X-ray absorptiometry (DXA) at 0, 12, and 24 months. Our aim was to inform the optimal exercise prescription for long-term prevention of obesity-related chronic diseases, such as postmenopausal breast cancer.

## 2. Materials and Methods

The methods and sample size calculation from the Breast Cancer and Exercise Trial in Alberta (BETA) were published previously [23, 25]. BETA was a two-armed, two-centre randomized (1 : 1) controlled exercise trial in healthy postmenopausal women in Calgary or Edmonton, Canada, conducted between June, 2010, and June, 2013. Besides dropouts, no intervention stopped early. Data collection for the 24-month follow-up started in June, 2012, and ended in May, 2014. Data were analyzed in 2017.

### 2.1. Study Sample

Participants were recruited through invitation letters from the Alberta Breast Screening Program and media campaigns. Eligibility was assessed by telephone. Inclusion criteria were as follows: age 50–74 years, postmenopausal, inactive (≤90 minutes/week moderate–vigorous activity), no previous cancer or major comorbidity, BMI 22–40 kg/m^2^, nonsmoker, nonexcessive alcohol use, nonhormone therapy user, and physician clearance for unrestricted physical activity. There was no racial or gender bias in the selection of participants. Potentially eligible participants were invited to an information session where they were informed about the 24-month follow-up. Free and informed consent was obtained in writing from all participants. The study protocol [23] was approved by the Alberta Cancer Research Ethics Committee and Conjoint Health Research Ethics Board at University of Calgary, and the Health Research Ethics Board at University of Alberta.

Study Coordinators in both cities randomly assigned participants to 150 or 300 minutes/week aerobic activity for 12 months. Randomization was stratified by the study centre and BMI with stratum-specific block sizes of four or six. The random allocation sequence was generated using R (version 3.0, R Foundation for Statistical Computing, Vienna, Austria) and user-defined functions. Staff unrelated to the study prepared numbered envelopes in which allocations were concealed. The research staff was blinded to a randomization group when taking body fat measurements.

### 2.2. Intervention

The intervention, described previously [23, 25], began with a three-month ramp up to 150 (moderate) or 300 (high) minutes/week activity, then continued with nine months of exercise maintenance. The intensity of the exercise prescription was 60–85% maximum heart rate reserve for both groups. Participants were asked to exercise under supervised conditions at a fitness facility for three days/week and on their own two days/week. Supervised sessions were held at the Westside Recreation Centre, Calgary or the Behavioural Medicine Fitness Centre, University of Alberta, Edmonton. Heart rate monitors (Polar FT4®, Polar Electro, Kempele, Finland) were worn during all exercise sessions. The duration, intensity, perceived exertion, and activity types were recorded by the exercise trainers in weekly exercise logs. There was no intervention from 12–24 months. At trial completion, participants were invited to a social event where they were given general tips for maintaining exercise but there were no specific instructions or requirements given to the participants to maintain their activity levels. They were aware that another full set of measurements would be taken at 24 months. The adherence to the year-long exercise intervention and during the 12-months postintervention has been previously reported [26].

### 2.3. Measures

Measurements were described previously [25]. In brief, physical fitness was assessed with a multistage, modified Balke submaximal cardiorespiratory treadmill test [27] at baseline, 6, 12, and 24 months; VO_2max_ estimation was previously described [25]. Anthropometric measurements were taken using a balance beam scale and stadiometer at baseline, 12 and 24 months in duplicate (if these measurements were not the same, then triplicate measurement was taken) by research staff and then averaged. Waist and hip circumferences were measured using the National Institutes for Health protocol [28]. Abdominal fat was measured from abdominal CT scans of four single slices centred at the umbilicus (the one most closely centred was selected for measurement by a radiologist) in Calgary using a Philips Brilliance Big Bore (Cleveland, Ohio, USA) or in Edmonton using a Toshiba Aquilion (Nasushiobara, Tochigi Prefecture, Japan). The study radiologist (AD) reviewed each scan and used image analysis software (Aquarius INtuition by TeraRecon, Inc.) to quantify subcutaneous and intra-abdominal fat. To measure total fat and lean mass, full-body DXA scans were taken in Calgary using a Hologic Discovery A DXA system (Bedford, MA, USA) and Hologic QDR software, or in Edmonton using a GE Healthcare Lunar Prodigy DXA (Madison, WI, USA) and GE Healthcare enCORE software, version 10.50. A multinational study found that the results for body composition were highly correlated between these two systems, with *r* values ranging from 0.96 to 0.98 [29].

Participants self-administered questionnaires at baseline, 12 and 24 months on health history and lifestyle, including past-year physical activities [30] and diet [31]. Metabolic equivalent of task (MET) values were assigned to each activity using the Compendium of Physical Activities [32]. Moderate–vigorous activity (MET-h/week) comprised the sum of MET-hours/week for all self-reported activities with MET values ≥3. Dietary energy intake (kcal/day) was estimated using diet ∗ calc (version 1.4.3, National Cancer Institute Applied Research Program, November, 2005).

Objective measurements of physical activity and sedentary behaviour were obtained with the ActiGraph GT3X + accelerometer (ActiGraph, LLC, Pensacola, FL, USA) and the activPAL3™ inclinometer (PAL Technologies Ltd., Glasgow, Scotland), respectively. Participants were asked to wear both devices during waking hours for seven days at baseline, 6, 12, and 24 months. Measurements were included if devices were worn ≥10 hrs/day and ≥4 days/session. ActiGraph data were downloaded using ActiLife software and grouped into 1-minute units. An objective measure of total physical activity was derived from the sum of ActiGraph measurements of all movement along the vertical axis using a cutoff of ≥100 counts/minute. ActivPAL3™ data were downloaded using activPAL3™ software, grouped into 1-minute units, and then summed to derive total sedentary time.

### 2.4. Statistical Analysis

Outcomes for the study were a variety of hypothesized biomarkers for postmenopausal breast cancer risk including total body fat, our primary outcome. In power calculations for the 24-month follow-up study, we expected 140 participants per group by 24 months. Assuming the 24-month effect sizes approximated the 12-month effect sizes of the intervention, we estimated that a two-sided, two-sample *t*-test would detect a between-arm difference in adiposity changes (0–24 months) equal to approximately one-third the standard deviation of the biomarker level (e.g., 4.2 kg of total body fat, 4.3 kg of weight, etc.), allowing for 5% Type I error and 80% power [33].

Participant characteristics between groups (high versus moderate; participants with versus without 24-month data) were compared using two-sample *t*-tests for continuous variables or *χ* ^2^ test for categorical variables. The primary analysis was intention-to-treat (among women with baseline and 24-month body fat measurements), to assess how group assignment affected 0–24 month body fat change. Participants with missing 24-month follow-up data were excluded. Least-squares mean differences in 0–24 month fat change between high and moderate groups were estimated from generalized linear models adjusted for baseline fat and study location (i.e., baseline to 24-month body fat change = *β*0 + *β*1 (intervention group) + *β*2 (body fat at baseline) + *β*3 (location)). For each participant, baseline weight and height were used to derive baseline BMI, 12-month measures to derive 12-month BMI, and 24-month measures to derive 24-month BMI. A post hoc analysis was conducted on 0–24 month change in height, to gauge potential impact on BMI results.

A sensitivity analysis was conducted, excluding seven participants who self-reported >1000 kcal/d change in energy intake between 0 and 12 months. Effect modification by baseline BMI, age (continuous variables), self-reported 12-month total physical activity (MET-hours/day), and change in self-reported total physical activity (MET-hours/day) between 0 and 12 months was assessed using statistical tests for interaction in linear models. Additionally, least-squares mean differences in 12–24 month fat change between high and moderate groups were estimated using generalized linear models adjusted for 12-month body fat and study location (i.e., 12–24 month body fat change = *β*0 + *β*1 (intervention group) + *β*2 (body fat at 12 months) + *β*3 (location)). Descriptive analyses explored 0–12 and 12–24 month changes in physical activity, sedentary behaviour, and dietary caloric intake as possible causes of fat change.

Exploratory analyses on 12–24 month fat changes were performed, stratifying participants by 0–12 month weight change. Specifically, weight change was treated as a categorical variable: “weight loss” was defined as losing ≥3% baseline weight, and “no weight loss” was losing <3% baseline weight or gaining weight [34]. We hypothesized greater fat regain in the high versus moderate group, specifically for women who experienced clinically significant weight loss. All analyses were performed using SAS (version 9.2, SAS Institute Inc., Cary, North Carolina, 2011). Graphics were produced using Microsoft Excel 2010. Statistical tests were two-sided with a 0.05 significance level.

## 3. Results

**Figure 1:**
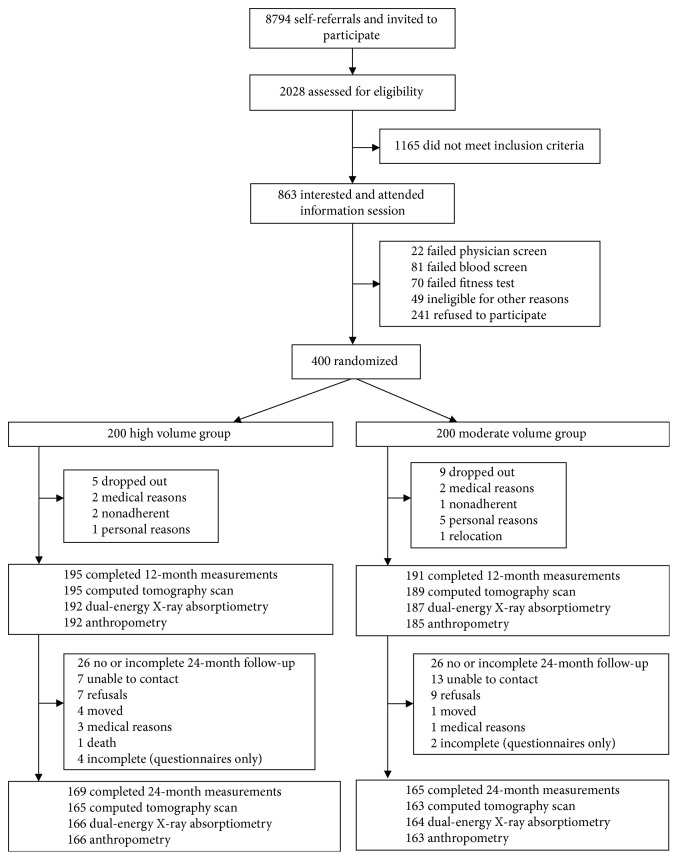
Flow of participants through BETA and the 24-month follow-up study, Alberta, Canada, 2010–2014.

Complete 24-month body fat data were available for 334 of 400 baseline participants (82.5% and 84.5% of moderate and high groups, respectively). Reasons for nonparticipation are shown in Figure 1. Compared to women with 24-month data, those without data were significantly younger at baseline (mean age 58.0 versus 59.7 years; *P* value = 0.02); less active during intervention (mean exercise time 0–12 months: 126 versus 180 minutes/week; *P* value < 0.01); and experienced smaller decreases in total fat (−0.89 versus −2.07 kg; *P* value = 0.04) and percent body fat (−0.6% versus −1.7%; *P* value = 0.02) (Table S1).

**Figure 2:**
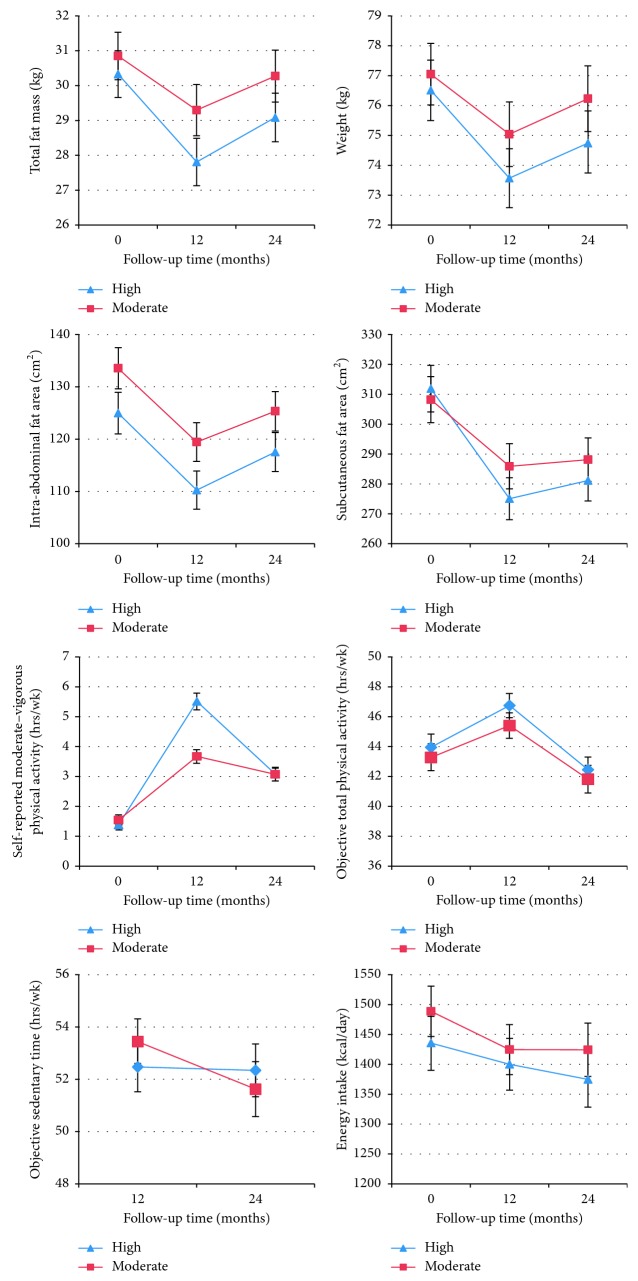
Average fat and lifestyle measurements over time for participants with data at all time points. Sample sizes based on data availability were as follows: *n*=163 high, *n*=159 moderate for body fat measures; *n*=167 high, *n*=162 moderate for self-reported moderate–vigorous physical activity; *n*=133 high, *n*=132 moderate for objectively measured total physical activity (ActiGraph, vertical axis measure); *n*=123 high, *n*=120 moderate for objectively measured sedentary time (activPAL™; data not collected at baseline); *n*=169 high, *n*=164 moderate for self-reported dietary energy intake.

Lifestyle behaviour changes are depicted in Figure 2. Although both groups showed decreased activity after the trial, the absolute decrease was greater for the high group. By 24 months, both groups were self-reporting practically the same amount of moderate–vigorous activity (high: 3.1 hours/week, 15.7 MET-hours/week; moderate: 3.1 hours/week, 15.4 MET-hours/week). By 24 months, the proportion of women achieving ≥200 minutes/week moderate–vigorous activity (recommended to prevent weight regain [12]) was 39% of the moderate group and 40% of the high group based on the self-report and 41% and 38%, respectively, from ActiGraph data. Both groups decreased sedentary time after the trial, by ∼1 h/week on average. Self-reported average energy intake remained relatively constant in both groups.

### 3.1. Baseline to 12 Months

Body composition changes during BETA (*n*=386) were published previously [23]. No serious adverse events were reported. Dropout rates during the trial were 2.5% and 4.5% for the high and moderate groups, respectively (Figure 1). Fifty-two BETA participants did not take part in the 24-month follow-up study. For women who did complete the 24-month follow-up (*n*=334, Table 1), baseline characteristics were similar to BETA participants [23].

### 3.2. Baseline to 24 Months

Body composition changes between 0 and 24 months are shown in Table 2. Between 0 and 24 months, total fat reduction was not significantly greater for the high versus moderate group (least-square mean change: −1.12 kg versus −0.42 kg, *P*=0.09; least-square mean difference, (high–moderate) = −0.70 kg, 95% CI: −1.50, 0.11). However, statistically significant dose effects were found for other measures; namely BMI (least-square mean difference, (high–moderate) = −0.42 kg/m^2^, 95% CI: 0.82, −0.02; *P*-value = 0.04), waist-to-hip ratio (−0.01, 95% CI: −0.02, −0.0002; *P*-value = 0.05), and subcutaneous abdominal fat area (−9.99, 95% CI: −19.52, −0.45 cm^2^; *P*-value = 0.04). Group differences in BMI change were consistent with those for body weight change (rather than height).

In exploratory analyses, the same regression models were stratified by BMI (<30, ≥30 kg/m^2^) and age (≤60, >60 years) to explore differential dose effects. Dose effects favouring the high arm were stronger in women with BMI ≥30 kg/m^2^ for all fat outcomes except the waist-to-hip ratio and percent body fat, although tests for interaction were not statistically significant (Table S2). When stratified by age, dose effects favouring the high arm were stronger in younger women for all fat outcomes except subcutaneous abdominal fat. Tests for interaction were not statistically significant (Table S3). We also assessed the effect modification by self-reported 12-month total physical activity (MET-hours/day) and change in self-reported total physical activity (MET-hours/day) between 0 and 12 months and found no statistical significance for any of the adiposity biomarkers. When *n*=7, women with extreme dietary caloric intake change (0–12 months) were excluded and dose effects were attenuated slightly except for the waist-to-hip ratio and lean mass (Table S4).

### 3.3. 12 Months to 24 Months

Exploratory analyses showed, on average, both high and moderate groups regained body fat between 12 and 24 months. Although the high group experienced larger fat increases than the moderate group, the between-group differences were not statistically significant (Table 3). Furthermore, there was no between-group difference in fat regain when the analysis was restricted to women who lost ≥3% of initial body weight during the trial (Table S5). Median weight regain as a proportion of weight lost was 34.0% in the moderate group and 41.1% in the high group. Figure 2 shows trajectories of average body fat at each time point; Figure S6 shows additional outcomes at each time point.

## 4. Discussion

This study showed that, in healthy postmenopausal women, average changes in total body fat from a high versus moderate exercise prescription were not significantly different between groups one year later. However, statistically significant, small dose effects were found for BMI, waist-to-hip ratio, and subcutaneous abdominal fat. Our primary results were similar across BMI and age categories. By 24 months, both high and moderate groups regained body fat and both groups self-reported ∼180 minutes/week moderate–vigorous physical activity on average.

To our knowledge, only one previous trial [24] tested how the dose of exercise prescribed impacts body fatness during follow-up. In that trial, 202 overweight adult men and women age 25–50 years were randomly assigned to 18 months of a high (2,500 kcal/week) or moderate (1,000 kcal/week) exercise volume (equivalent to walking ∼30 minutes/day or ∼75 minutes/day, respectively [24]) concurrently with behavioural therapy group sessions for obesity. Twelve months after intervention, there was still greater weight loss in the high-volume group, though not statistically significantly (−2.86 kg versus −0.9 kg weight loss from baseline, *P*-value = 0.16). The study did not measure changes in subcutaneous or intra-abdominal fat. Similarly, a large observational study of National Weight Registry participants (*n*=3,591) examined current exercise dose in relation to future weight maintenance. After three years of follow-up, weight regain was similar irrespective of baseline activity (∼moderate–intensity <30 minutes/day, 30–60 minutes/day, 60–90 minutes/day, ≥90 minutes/day) [35]. However, this study is less comparable to ours because it focused on adults who already successfully maintained substantial weight loss, ≥13.6 kg (30 lbs).

Follow-up studies have also been done on exercise trials that did not randomize by dose (or intervene on diet). On average, relative to baseline, these studies showed fat loss maintained at one year [36] or after one month of detraining [37]; interventions were 240 minutes/week with mild caloric restriction [36] or 130 minutes/week [37] of mainly aerobic exercise, respectively. Other studies showed partial fat regain after one year [38], three months [39], or one month of detraining [40]; interventions were 150–225 minutes/week aerobic/resistance training [38], 135 minutes/week aerobic/resistance training [39], and three times/week resistance training [40], respectively. Our findings are generally consistent with these studies, showing some sustained benefit from an aerobic intervention but also partial regain.

We are unaware of any dose-response exercise trials that measured intra-abdominal (visceral) fat changes postintervention. Visceral fat is of etiological interest, given its adverse association with cardiovascular disease risk factors, insulin resistance [7], and possibly cancer [8]. In 101 Japanese women, Koga et al. reported that greater daily fluctuation in exercise (standard deviation, minutes/week) during a weight loss intervention was associated with greater regain of visceral fat one year later [41]. If exercise consistency is important for lowering visceral fat, this might explain no dose effect in our study since participants were randomized to different exercise durations, not frequency. The visceral fat regain we observed was also reported by Hunter et al., who related regain to physical inactivity (38% regain among women who did not adhere to a year-long, postweight loss exercise program versus < 0.8% regain in adherers [42]).

A behaviour that likely contributed to regain in our study was decreased physical activity between 12 and 24 months. Average self-reported physical activity decreased in both groups between 12 and 24 months, and by 24 months they were similar, ∼180 minutes/week moderate–vigorous activity. These comparable activity levels at 24 months likely arose since many of the moderate group participants wanted to exercise more than 150 minutes/week during the study and then without the constraints imposed by the trial, they were able to increase their activity levels during the 12-month follow-up period. Likewise, the high-group participants had more difficulty achieving and maintaining the higher exercise prescription during the trial and, consequently, after the trial, they reduced their activity levels to 3.1 hours/week which was more sustainable for them. Similarly, Tate et al. [24] showed that 12 months postintervention, average exercise levels did not differ significantly between groups (1,696 versus 1,390 kcal/week for high versus moderate dose, *P*-value > 0.10). To maintain fat loss, 200 minutes/week moderate-intensity activity may be required [12]. In our own data at 24 months, the proportion of women achieving ≥200 minutes/week was nearly identical in the two arms (39% and 40%), perhaps explaining no dose effect for some outcomes.

There are important distinctions between our study and previous postintervention studies with fat outcomes [24, 36–40]. First, we examined long-term changes in whole-body fat and abdominal fat, not only weight loss. Second, this study did not include a comprehensive weight loss intervention (comprising diet, exercise, and behavioural counselling), but rather an exercise-only intervention. Third, we studied the real-world implications of our intervention using an observational follow-up, unlike other studies with long interventions [15, 43]. Fourth, we measured postintervention body fat unlike another similar trial in postmenopausal women [11]. Other strengths include a randomized controlled trial design that eliminated potential confounding, an intense exercise prescription, sophisticated body fat measures, and a relatively large sample size.

Our results may be most generalizable to healthy postmenopausal women who are overweight, inactive, and around age 60. There was some evidence of selection bias in our study. Three-hundred twenty-nine out of 400 eligible participants were analyzed, with greater representation from women with better adherence during the trial. The less-adherent population might experience different fat changes, perhaps with less fat regain. A limitation of this study was our inability to disentangle metabolic versus behavioural influences on fat regain; a detraining study would help in this regard. Rather, our intent was to identify persistent dose effects after the intervention. Another limitation may have been insufficient statistical power with which dose effects are detected, particularly in subgroup analyses and exploratory analyses.

## 5. Conclusion

We found that, for some fat measures, small exercise dose effects were still evident 12 months after intervention, despite similar exercise levels during follow-up. Twenty-four month fat loss was greater in the high- versus moderate-duration exercise group by 0.42 kg/m^2^ for BMI, 0.01 for waist-to-hip ratio, and 10 cm^2^ for subcutaneous abdominal fat. However, the clinical significance of these differences is unclear, and there was no significant group difference in total fat loss. Therefore, interventions prescribing longer durations of exercise beyond 150 minutes/week–to inactive, postmenopausal women may have small effects for enhancing body fat reductions over the long-term and more exercise and/or reduced caloric intake may be needed.

## Figures and Tables

**Table 1:** Baseline characteristics and 0–12 month changes for participants with 24-month body fat data.

Characteristic	Moderate	High
*Baseline measurements* ^*a*^		
*N*	165	169
Married or common law, no. (%)	113 (68.5)	119 (70.4)
Educated beyond high school, no. (%)	128 (77.6)	135 (79.9)
Employed full time, no. (%)	50 (30.3)	58 (34.3)
Ethnicity (white), no. (%)	152 (92.1)	145 (85.8)
Age (y)	59.8 (5.1)	59.7 (5.0)
Weight (kg)	77.1 (12.8)	76.7 (12.8)
Height (m)	1.62 (0.06)	1.63 (0.06)
Body mass index (kg/m^2^)	29.4 (4.4)	28.9 (4.4)
Maximum oxygen consumption, VO_2max_ (mL/kg/min)	27.0 (4.9)	26.6 (5.4)
Total physical activity (MET-h/week)^b^	95.6 (48.2)	94.2 (45.0)
Recreational physical activity (MET-h/week)^b^	9.8 (13.6)	9.4 (9.6)
Moderate–vigorous physical activity (MET-h/week)^b^	7.3 (12.2)	6.9 (9.4)
Total energy intake (kcal/d)^b^	1496 (535)	1434 (596)
*0–12 month change, mean (SD)* ^*c*^		
Total physical activity (MET-h/week)^b^	18.5 (50.2)	27.4 (50.1)
Recreational physical activity (MET-h/week)^b^	14.2 (17.4)	27.1 (20.1)
Moderate–vigorous physical activity (MET-h/week)^b^	14.1 (18.0)	27.4 (20.1)
Total energy intake (kcal/d)^b^	−70.4 (374.6)	−35.8 (400.6)
Weight (kg)	−2.0 (3.9)	−2.9 (4.6)
Height (cm)	0.13 (1.1)	0.31 (1.2)
Body mass index (kg/m^2^)	−0.8 (1.5)	−1.2 (1.8)
Waist circumference (cm)	−5.0 (5.6)	−6.4 (7.6)
Hip circumference (cm)	−2.2 (4.1)	−2.6 (5.1)
Waist-to-hip ratio	−0.028 (0.044)	−0.038 (0.055)
Total body fat (kg)	−1.54 (3.20)	−2.59 (4.08)
Percent body fat (%)^d^	−1.2 (2.6)	−2.2 (3.6)
Lean mass (kg)	−0.36 (1.75)	−0.16 (1.84)
Subcutaneous abdominal fat area (cm^2^)	−22.7 (39.0)	−36.6 (47.9)
Intra-abdominal fat area (cm^2^)	−14.2 (23.5)	−14.7 (23.5)
Total abdominal fat area (cm^2^)	−36.8 (54.3)	−51.3 (61.9)

^a^ None of the group differences at baseline were statistically significant. ^b^ Derived from self-report. ^c^ Detailed results for the 0–12 month body fat changes for the entire study population were reported in Friedenreich et al. [23]. The results in this table pertain to 334 participants with body fat data at the 24-month follow-up. ^d^ Percent body fat was calculated as 100% × (fat mass/(fat mass + lean mass)).

**Table 2:** Body composition changes (0–24 months) in the Breast Cancer and Exercise Trial in Alberta, 2010–2014.

		Moderate		high		
Body measure	*n* ^a^	LS mean change (95% CI)^b^	*n* ^a^	LS mean change (95% CI)^b^	Between-group difference, high–moderate (95% CI)^b^	*P* value^c^
Total fat mass (kg)	164	−0.42 (−1.02 to 0.19)	166	−1.12 (−1.72 to −0.51)	−0.70 (−1.50 to 0.11)	0.09
Weight (kg)	163	−0.79 (−1.57 to −0.02)	165	−1.81 (−2.58 to −1.03)	−1.02 (−2.04 to 0.01)	0.05
Height (cm)	163	−0.14 (−0.32 to 0.05)	165	0.01 (−0.17 to 0.20)	0.15 (−0.10 to 0.40)	0.23
Body mass index (kg/m^2^)	163	−0.25 (−0.55 to 0.05)	165	−0.66 (−0.97 to −0.36)	**−0.42 (−0.82 to −0.02)**	**0.04**
Waist circumference (cm)	163	−3.73 (−4.82 to −2.64)	165	−5.00 (−6.09 to −3.90)	−1.26 (−2.72 to 0.19)	0.09
Hip circumference (cm)	163	−1.56 (−2.32 to −0.79)	165	−1.70 (−2.47 to −0.93)	−0.14 (−1.16 to 0.87)	0.78
Waist-to-hip ratio	163	−0.023 (−0.030 to −0.016)	165	−0.033 (−0.040 to −0.026)	**−0.010 (−0.019 to −0.0002)**	0.05
Total lean mass (kg)	164	−0.22 (−0.50 to 0.06)	166	−0.18 (−0.46 to 0.10)	0.05 (−0.33 to 0.42)	0.81
Percent body fat (%)	164	−0.40 (−0.89 to 0.08)	166	−0.98 (−1.47 to −0.50)	−0.58 (−1.22 to 0.06)	0.08
Subcutaneous abdominal fat area (cm^2^)	163	−22.20 (−29.34 to −15.05)	166	−32.18 (−39.30 to −25.06)	**−9.99 (−19.52 to −0.45)**	**0.04**
Intra-abdominal fat area (cm^2^)	163	−7.35 (−11.12 to −3.58)	166	−8.04 (−11.81 to −4.27)	−0.70 (−5.75 to 4.36)	0.79
Total abdominal fat area (cm^2^)	163	−29.43 (−39.11 to −19.75)	166	−40.42 (−50.08 to −30.75)	−10.99 (−23.93 to 1.95)	0.10

^a^ Number of women completing adiposity measures at baseline and 24-month follow-up, for whom a change could be calculated, within each randomization group. ^b^ Least-square group mean of the high and moderate exercise groups and their between-group difference were estimated from general linear model specified as body fat change = *β*0 + *β*1 (intervention group) + *β*2 (body fat at baseline) + *β*3 (location). Measurements at 12 months were ignored. ^c^ *P* value for the test of significance for the null hypothesis that the LS mean difference between the two intervention groups equals 0. Boldface indicates statistical significance (*P* < 0.05 or a 95% confidence interval that does not include zero).

**Table 3:** Body composition changes (12–24 months) in the Breast Cancer Exercise Trial in Alberta, 2010–2014.

		Moderate		high		
Body fat measure	*n* ^a^	LS mean change (95% CI)^b^	*n* ^a^	LS mean change (95% CI)^b^	Between-group difference, high–mod (95% CI)^b^	*P* value^c^
Weight (kg)	159	0.97 (0.30 to 1.64)	163	0.90 (0.23 to 1.57)	−0.08 (−0.96 to 0.81)	0.87
Body mass index (kg/m^2^)	159	0.45 (0.20 to 0.71)	163	0.45 (0.20 to 0.71)	0.003 (−0.33 to 0.34)	0.98
Waist circumference (cm)	159	0.86 (−0.16 to 1.87)	163	1.25 (0.24 to 2.27)	0.40 (−0.95 to 1.74)	0.56
Hip circumference (cm)	159	0.52 (−0.20 to 1.23)	163	0.79 (0.08 to 1.50)	0.27 (−0.67 to 1.22)	0.57
Waist-to-hip ratio	159	0.004 (−0.004 to 0.011)	163	0.003 (−0.004 to 0.011)	−0.0003 (−0.010 to 0.009)	0.96
Total lean mass (kg)	162	0.01 (−0.22 to 0.25)	164	−0.16 (−0.39 to 0.07)	−0.18 (−0.49 to 0.14)	0.27
Total fat mass (kg)	162	1.12 (0.60 to 1.64)	164	1.35 (0.83 to 1.87)	0.23 (−0.47 to 0.93)	0.52
Percent body fat (%)	162	1.05 (0.61 to 1.49)	164	1.26 (0.82 to 1.69)	0.22 (−0.37 to 0.82)	0.49
Subcutaneous abdominal fat area (cm^3^)	163	1.14 (−5.29 to 7.57)	166	3.41 (−3.02 to 9.85)	2.27 (−6.34 to 10.89)	0.60
Intra-abdominal fat area (cm^2^)	163	5.37 (1.85 to 8.88)	166	5.85 (2.34 to 9.36)	0.48 (−4.23 to 5.19)	0.84
Total abdominal fat area (cm^2^)	163	6.67 (−1.88 to 15.22)	166	9.11 (0.55 to 17.67)	2.44 (−9.02 to 13.91)	0.68

^a^ Number of women completing measures at follow-up and end of study, for whom a change could be calculated, within each randomization group. ^b^ Least-square group mean of the high and moderate exercise groups and their between-group difference were estimated from general linear model specified as body fat change = *β*0 + *β*1 ∗ (intervention group) + *β*2 ∗ (body fat at 12 months) + *β*3 ∗ (location). ^c^ *P* value for the test of significance for the null hypothesis that the LS mean difference between the two intervention groups equals 0.

## Data Availability

The dataset analyzed during the current study is available from the corresponding author on reasonable request.

## Abbreviations

BETA: Breast Cancer and Exercise Trial in Alberta
BMI: Body mass index
CT: Computed tomography
DXA: Dual-energy X-ray absorptiometry
HIGH: High-duration group, prescribed 300 minutes/week of exercise
MET: Metabolic equivalent of task
MOD or moderate: Moderate-duration group, prescribed 150 minutes/week of exercise.

## Supplementary Materials

Supplementary Materials Supplementary Tables Table S1: comparison of participant characteristics and intervention changes between participants and nonparticipants in the 24-month follow-up study. Table S2: body composition changes between 0 and 24 months stratified by body mass index in the Breast Cancer and Exercise Trial in Alberta, 2010–2014. Table S3: body composition changes between 0 and 24 months stratified by age in the Breast Cancer and Exercise Trial in Alberta, 2010–2014. Table S4: sensitivity analysis of body composition changes (0–24 months) for participants in the Breast Cancer and Exercise Trial in Alberta, 2010–2014. Table S5: postintervention body composition changes (12–24 months) according to weight change during the Breast Cancer and Exercise Trial in Alberta, 2010–2014. Figure S6: additional average measurements over time for participants with complete data at each time point. Sample sizes based on data availability were *n*=163 high and *n*=159 moderate. Abbreviations: BMI, body mass index (kg/m^2^); WHR, waist-to-hip ratio.
