# Supplementary material for: Exercise Dose Effects on Body Fat 12 Months after an Exercise Intervention: Follow-up from a Randomized Controlled Trial
Source: J Obes. 2019 Jan 20;2019:3916416. doi: 10.1155/2019/3916416 (PMC6363238; doi:10.1155/2019/3916416)
Supplement: Supplementary Materials — Supplementary Tables Table S1: comparison of participant characteristics and intervention changes between participants and nonparticipants in the 24-month follow-up study. Table S2: body composition changes between 0 and 24 months stratified by body mass index in the Breast Cancer and Exercise Trial in Alberta, 2010–2014. Table S3: body composition changes between 0 and 24 months stratified by age in the Breast Cancer and Exercise Trial in Alberta, 2010–2014. Table S4: sensitivity analysis of body composition changes (0–24 months) for participants in the Breast Cancer and Exercise Trial in Alberta, 2010–2014. Table S5: postintervention body composition changes (12–24 months) according to weight change during the Breast Cancer and Exercise Trial in Alberta, 2010–2014. Figure S6: additional average measurements over time for participants with complete data at each time point. Sample sizes based on data availability were n=163 high and n=159 moderate. Abbreviations: BMI, body mass index (kg/m2); WHR, waist-to-hip ratio. [file 3916416.f1.docx]

**Supplementary Tables**

**Table S1:** Comparison of participant characteristics and intervention changes between participants and non-participants in the 24-month follow-up study

|  | **Non-Participants ^a^** | **Participants ^b^** | ***P*-value ^c^** |
| --- | --- | --- | --- |
| **Baseline characteristics** |  |  |  |
| N | 52 | 334 |  |
| BETA group assignment (no. HIGH /MOD) | 26/26 | 169/165 |  |
| Married or common law, no. (%) | 31 (59.6) | 232 (69.5) | 0.16 |
| Educated beyond high school, no. (%) | 39 (75.0) | 263 (78.7) | 0.54 |
| Employed full time, no. (%) | 14 (26.9) | 108 (32.3) | 0.43 |
| Ethnicity | 47 (90.4) | 297 (88.9) | 0.75 |
| Age, mean (SD), y | 58.0 (4.4) | 59.7 (5.0) | 0.02 |
| Weight, mean (SD), kg | 79.2 (14.3) | 76.9 (12.8) | 0.23 |
| Body mass index, mean (SD) | 29.9 (4.4) | 29.1 (4.4) | 0.23 |
| Maximum oxygen consumption, VO_2max_, mean (SD), mL/kg/min | 26.6 (4.5) | 26.8 (5.2) | 0.76 |
| Total physical activity, mean (SD), MET-h/week | 98.3 (39.6) | 94.9 (46.6) | 0.61 |
| Recreational physical activity, mean (SD), MET-h/week | 8.9 (10.0) | 9.6 (11.8) | 0.67 |
| **0-12 months** |  |  |  |
| Average exercise time in BETA, mean (SD), minutes/week | 125.7 (84.8) | 180.1 (81.6) | < 0.01 |
| Recreational physical activity change, mean (SD), MET-h/week | 17.5 (18.0) | 20.8 (19.9) | 0.31 |
| Total physical activity change, mean (SD), MET-h/week | 12.5 (53.9) | 23.0 (50.3) | 0.19 |
| Moderate-vigorous physical activity change, mean (SD), MET-h/week | 18.6 (18.5) | 20.8 (20.2) | 0.49 |
| Body weight change, mean (SD), kg | -1.3 (4.9) | -2.4 (4.3) | 0.08 |
| Body mass index change, mean (SD), | -0.6 (1.8) | -1.0 (1.7) | 0.13 |
| Waist circumference change, mean (SD), cm | -3.8 (5.8) | -5.7 (6.7) | 0.06 |
| Hip circumference change, mean (SD), cm | -1.9 (5.4) | -2.4 (4.7) | 0.46 |
| Waist-to-hip ratio change | -0.021 (0.050) | -0.033 (0.050) | 0.12 |
| Total body fat change, mean (SD), kg | -0.89 (3.55) | -2.07 (3.70) | 0.04 |
| Percent fat change, mean (SD), % | -0.6 (2.7) | -1.7 (3.2) | 0.02 |
| Lean mass change, mean (SD), kg | -0.36 (2.59) | -0.26 (1.80) | 0.79 |
| Subcutaneous abdominal fat area change, mean (SD), cm^2^ | -19.4 (56.0) | -29.7 (44.2) | 0.22 |
| Intra-abdominal fat area change, mean (SD), cm^2^ | -7.9 (20.6) | -14.4 (23.4) | 0.06 |
| Total abdominal fat area change, mean (SD), cm^2^ | -27.3 (71.9) | -44.2 (58.6) | 0.12 |

^a^ Women who completed intervention but did not complete the 24-month adiposity measurement

^b^ Women who completed intervention and participated in 24-month follow-up

^c^ P value for the test of significance that the difference between the mean of MOD and HIGH group equals zero, by Student’s t-test

BETA, Breast Cancer and Exercise Trial in Alberta.

**Table S2:** Body composition changes between 0-24 months stratified by body mass index in the Breast Cancer and Exercise Trial in Alberta, 2010-2014

|  |  | **Moderate Volume** | | **High Volume** | |  |  |
| --- | --- | --- | --- | --- | --- | --- | --- |
| **Adiposity measure** | **BMI** | **n ^a^** | **LS Mean Change (95% CI) ^b^** | **n ^a^** | **LS Mean Change (95% CI) ^b^** | **Between-Group Difference, HIGH-MOD (95% CI) ^b^** | ***P* for interaction ^c^** |
| Body weight, kg | < 30 | 104 | -0.74 (-1.53 to 0.05) | 105 | -1.13 (-1.92 to -0.33) | -0.39 (-1.43 to 0.65) | 0.11 |
|  | ≥ 30 | 59 | -0.89 (-2.52 to 0.75) | 60 | -2.96 (-4.59 to -1.34) | -2.08 (-4.27 to 0.11) |  |
| Body mass index, kg/m^2^ | < 30 | 104 | -0.23 (-0.54 to 0.09) | 105 | -0.43 (-0.74 to -0.11) | -0.20 (-0.62 to 0.21) | 0.20 |
|  | ≥ 30 | 59 | -0.32 (-0.96 to 0.31) | 60 | -1.06 (-1.68 to -0.43) | -0.73 (-1.58 to 0.12) |  |
| Waist circumference, cm | < 30 | 104 | -3.60 (-4.87 to -2.33) | 105 | -4.67 (-5.94 to -3.39) | -1.07 (-2.74 to 0.61) | 0.94 |
|  | ≥ 30 | 59 | -4.00 (-6.00 to -2.00) | 60 | -5.61 (-7.60 to -3.62) | -1.62 (-4.29 to 1.06) |  |
| Hip circumference, cm | < 30 | 104 | -1.63 (-2.41 to -0.85) | 105 | -1.54 (-2.33 to -0.75) | 0.09 (-0.94 to 1.12) | 0.84 |
|  | ≥ 30 | 59 | -1.19 (-2.80 to 0.42) | 60 | -2.01 (-3.59 to -0.44) | -0.82 (-2.96 to 1.31) |  |
| Waist-to-hip ratio | < 30 | 104 | -0.022 (-0.031 to -0.013) | 105 | -0.033 (-0.042 to -0.024) | -0.011 (-0.023 to 0.001) | 0.79 |
|  | ≥ 30 | 59 | -0.026 (-0.039 to -0.014) | 60 | -0.033 (-0.045 to -0.021) | -0.007 (-0.023 to 0.010) |  |
| Total lean mass, kg | < 30 | 105 | -0.06 (-0.38 to 0.26) | 104 | 0.08 (-0.24 to 0.40) | 0.14 (-0.28 to 0.56) | 0.30 |
|  | ≥ 30 | 59 | -0.52 (-1.06 to 0.02) | 62 | -0.67 (-1.21 to -0.14) | -0.15 (-0.87 to 0.56) |  |
| Total fat mass, kg | < 30 | 105 | -0.44 (-1.05 to 0.17) | 104 | -0.94 (-1.56 to -0.32) | -0.50 (-1.61 to 0.32) | 0.52 |
|  | ≥ 30 | 59 | -0.35 (-1.65 to 0.94) | 62 | -1.42 (-2.67 to -0.16) | -1.06 (-2.79 to 0.66) |  |
| Percent body fat, % | < 30 | 105 | -0.46 (-1.07 to 0.15) | 104 | -1.08 (-1.69 to -0.47) | -0.62 (-1.42 to 0.18) | 0.68 |
|  | ≥ 30 | 59 | -0.01 (-0.84 to 0.81) | 62 | -0.62 (-1.42 to 0.17) | -0.61 (-1.69 to 0.46) |  |
| Subcutaneous abdominal fat area, cm^2^ | < 30 | 104 | -18.98 (-28.02 to -9.94) | 104 | -24.79 (-33.91 to -15.67) | -5.81 (-17.84 to 6.21) | 0.29 |
|  | ≥ 30 | 59 | -29.83 (-40.64 to -19.01) | 62 | -46.82 (-57.37 to -36.28) | -17.00 (-31.43 to -2.56) |  |
| Intra-abdominal fat area, cm^2^ | < 30 | 104 | -5.28 (-9.62 to -0.94) | 104 | -2.51 (-6.90 to 1.89) | 2.77 (-3.06 to 8.61) | 0.18 |
|  | ≥ 30 | 59 | -11.64 (-18.73 to -4.56) | 62 | -17.21 (-24.16 to -10.25) | -5.56 (-15.05 to 3.93) |  |
| Total fat area, cm^2^ | < 30 | 104 | -23.66 (-35.72 to -11.60) | 104 | -27.45 (-39.68 to -15.22) | -3.78 (-19.90 to 12.33) | 0.20 |
|  | ≥ 30 | 59 | -42.78 (-57.66 to -27.91) | 62 | -65.96 (-80.50 to -51.42) | -23.18 (-43.01 to -3.35) |  |

^a^ Number of participants completing measures at baseline and 24 months, within each randomization group

^b^ Least-square mean change from 0 to 24 months and LS mean difference between the two intervention groups, estimated from generalized linear model

^c^ P refers to the statistical significance of the interaction term between the high volume exercise group and the stratified variable, with the model as: adiposity change = β0 + β1 × group + β2 × baseline adiposity + β3 × location + β4 × baseline BMI + β5 × baseline BMI ×group, where BMI was treated as a continuous covariate.

**Table S3:** Body composition changes between 0-24 months stratified by age in the Breast Cancer and Exercise Trial in Alberta, 2010-2014

|  |  | **Moderate Volume** | | **High Volume** | |  |  |
| --- | --- | --- | --- | --- | --- | --- | --- |
| **Adiposity measure** | **Age** | **n ^a^** | **LS Mean Change (95% CI) ^b^** | **n ^a^** | **LS Mean Change (95% CI) ^b^** | **Between-Group Difference, HIGH-MOD (95% CI) ^b^** | ***P* for interaction ^c^** |
| Body weight, kg | ≤ 60 | 88 | -0.10 (-1.18 to 0.99) | 97 | -1.94 (-3.02 to -0.87) | -1.85 (-3.32 to -0.38) | 0.29 |
|  | > 60 | 75 | -1.63 (-2.74 to -0.51) | 68 | -1.70 (-2.81 to -0.59) | -0.07 (-1.49 to 1.35) |  |
| Body mass index, kg/m^2^ | ≤ 60 | 88 | 0.03 (-0.40 to 0.45) | 97 | -0.71 (-1.13 to -0.29) | -0.74 (-1.31 to -0.16) | 0.24 |
|  | > 60 | 75 | -0.58 (-1.01 to -0.15) | 68 | -0.62 (-1.05 to -0.19) | -0.05 (-0.60 to 0.50) |  |
| Waist circumference, cm | ≤ 60 | 88 | -3.00 (-4.50 to -1.51) | 97 | -5.53 (-7.01 to -4.05) | -2.53 (-4.56 to -0.51) | 0.22 |
|  | > 60 | 75 | -4.63 (-6.28 to -2.97) | 68 | -4.30 (-5.94 to -2.65) | 0.33 (-1.77 to 2.43) |  |
| Hip circumference, cm | ≤ 60 | 88 | -1.05 (-2.15 to 0.05) | 97 | -1.81 (-2.89 to -0.72) | -0.76 (-2.25 to 0.73) | 0.63 |
|  | > 60 | 75 | -2.37 (-3.43 to -1.30) | 68 | -1.63 (-2.69 to -0.57) | 0.74 (-0.61 to 2.09) |  |
| Waist-to-hip ratio | ≤ 60 | 88 | -0.021 (-0.030 to -0.011) | 97 | -0.036 (-0.046 to -0.027) | -0.016 (-0.029 to -0.003) | 0.28 |
|  | > 60 | 75 | -0.025 (-0.036 to -0.014) | 68 | -0.028 (-0.039 to -0.017) | -0.003 (-0.018 to 0.011) |  |
| Total lean mass, kg | ≤ 60 | 88 | 0.06 (-0.31 to 0.43) | 100 | -0.13 (-0.49 to 0.22) | -0.20 (-0.69 to 0.30) | 0.50 |
|  | > 60 | 76 | -0.48 (-0.92 to -0.05) | 66 | -0.29 (-0.74 to 0.17) | 0.19 (-0.37 to 0.76) |  |
| Total fat mass, kg | ≤ 60 | 88 | 0.02 (-0.84 to 0.87) | 100 | -1.19 (-2.02 to -0.36) | -1.21 (-2.36 to -0.06) | 0.46 |
|  | > 60 | 76 | -1.06 (-1.92 to -0.19) | 66 | -1.08 (-1.97 to -0.20) | -0.03 (-1.14 to 1.09) |  |
| Percent body fat, % | ≤ 60 | 88 | -0.20 (-0.87 to 0.47) | 100 | -0.97 (-1.61 to -0.33) | -0.76 (-1.66 to 0.13) | 0.87 |
|  | > 60 | 76 | -0.74 (-1.47 to -0.02) | 66 | -1.09 (-1.84 to -0.34) | -0.35 (-1.27 to 0.58) |  |
| Subcutaneous abdominal fat area, cm^2^ | ≤ 60 | 88 | -23.63 (-34.11 to -13.15) | 97 | -33.01 (-43.29 to -22.72) | -9.37 (-23.59 to 4.84) | 0.89 |
|  | > 60 | 75 | -20.26 (-29.88 to -10.64) | 69 | -30.78 (-40.35 to -21.21) | -10.52 (-22.87 to 1.83) |  |
| Intra-abdominal fat area, cm^2^ | ≤ 60 | 88 | -5.64 (-10.85 to -0.42) | 97 | -8.34 (-13.46 to -3.21) | -2.70 (-9.79 to 4.39) | 0.26 |
|  | > 60 | 75 | -9.48 (-15.14 to -3.81) | 69 | -7.54 (-13.21 to -1.87) | 1.94 (-5.37 to 9.24) |  |
| Total fat area, cm^2^ | ≤ 60 | 88 | -29.05 (-43.33 to -14.78) | 97 | -41.60 (-55.63 to -27.57) | -12.55 (-31.93 to 6.84) | 0.59 |
|  | > 60 | 75 | -29.62 (-42.44 to -16.80) | 69 | -38.22 (-51.01 to -25.43) | -8.60 (-25.08 to 7.88) |  |

^a^ Number of participants completing measures at baseline and 24 months, within each randomization group

^b^ Least-square mean change from 0 to 24 months and LS mean difference between the two intervention groups, estimated from generalized linear model

^c^ P refers to the statistical significance of the interaction term between the high volume exercise group and the stratified variable, with the model as: adiposity change = β0 + β1 × group + β2 × baseline adiposity + β3 × location + β4 × baseline Age + β5 × baseline Age ×group, where BMI was treated as a continuous covariate.

**Table S4:** Sensitivity analysis of body composition changes (0-24 months) for participants in the Breast Cancer and Exercise Trial in Alberta, 2010-2014

|  | **Primary analysis ^a^**  **(N=334)** | | **Sensitivity analysis removing dietary outliers ^b^ (N=327)** | |
| --- | --- | --- | --- | --- |
| **Adiposity measure** | **Between-Group Difference, HIGH-MOD (95% CI) ^b^** | ***P*-value for dose effects, 0-24 month change** | **Between-Group Difference, HIGH-MOD (95% CI) ^b^** | ***P*-value for dose effects, 0-24 month change** |
| Body weight, kg | -1.02 (-2.04 to 0.01) | 0.05 | -0.90 (-1.92 to 0.12) | 0.08 |
| Body mass index, kg/m^2^ | -0.42 (-0.82 to -0.02) | 0.04 | -0.38 (-0.78 to 0.02) | 0.06 |
| Waist circumference, cm | -1.26 (-2.72 to 0.19) | 0.09 | -1.07 (-2.53 to 0.40) | 0.15 |
| Hip circumference, cm | -0.14 (-1.16 to 0.87) | 0.78 | 0.10 (-0.91 to 1.12) | 0.84 |
| Waist-to-hip ratio | -0.010  (-0.019 to -0.0002) | 0.05 | -0.010  (-0.020 to -0.0001) | 0.05 |
| Total lean mass, kg | 0.05 (-0.33 to 0.42) | 0.81 | 0.08 (-0.29 to 0.45) | 0.66 |
| Total fat mass, kg | -0.70 (-1.50 to 0.11) | 0.09 | -0.61 (-1.41 to 0.20) | 0.14 |
| Percent body fat, % | -0.58 (-1.22 to 0.06) | 0.08 | -0.51 (-1.15 to 0.13) | 0.12 |
| Subcutaneous abdominal fat area, cm^2^ | -9.99  (-19.52 to -0.45) | 0.04 | -8.49  (-18.02 to 1.05) | 0.08 |
| Intra-abdominal fat area, cm^2^ | -0.70 (-5.75 to 4.36) | 0.79 | -0.50 (-5.59 to 4.60) | 0.85 |
| Total abdominal fat area, cm^2^ | -10.99  (-23.93 to 1.95) | 0.10 | -9.27  (-22.19 to 3.66) | 0.16 |

^a^ Results shown in Table 2. *P-*value for the test of significance to the null hypothesis that the LS mean difference between the two intervention groups equals 0 against the two-sided alternative hypothesis.

^b^ Seven participants were excluded who completed 24 months follow-up who self-reported > 1,000 kcal/d change in energy intake during the intervention period, from 0-12 months.

**Table S5:** Post-intervention body composition changes (12-24 months) according to weight change ^a^ during the Breast Cancer and Exercise Trial in Alberta, 2010-2014

|  |  | **12-24 month change ^b^** | | | |  |
| --- | --- | --- | --- | --- | --- | --- |
| **Biomarker** | **0-12 month change ^a^** | **MODERATE** | | **HIGH** | |  |
|  |  | **N** | **Mean (95% CI)** | **N** | **Mean (95% CI)** | ***P*-value ^c^** |
| Body weight, kg | Weight loss | 71 | 1.93 (1.00 to 2.86) | 82 | 2.07 (1.04 to 3.09) | 0.85 |
|  | No weight loss | 88 | 0.59 (-0.22 to 1.39) | 81 | 0.27 (-0.49 to 1.04) | 0.58 |
| Body mass index, kg/m^2^ | Weight loss | 71 | 0.68 (0.33 to 1.03) | 82 | 0.83 (0.44 to 1.23) | 0.58 |
|  | No weight loss | 88 | 0.26 (-0.04 to 0.57) | 81 | 0.13 (-0.15 to 0.40) | 0.51 |
| Total body fat, kg | Weight loss | 71 | 1.59 (0.81 to 2.37) | 83 | 2.04 (1.35 to 2.73) | 0.39 |
|  | No weight loss | 88 | 0.51 (-0.19 to 1.21) | 79 | 0.47 (-0.17 to 1.11) | 0.93 |
| Percent body fat, % | Weight loss | 71 | 1.18 (0.51 to 1.86) | 83 | 1.73 (1.09 to 2.38) | 0.24 |
|  | No weight loss | 88 | 0.37 (-0.21 to 0.94) | 79 | 0.41 (-0.13 to 0.95) | 0.92 |
| Total lean mass, kg | Weight loss | 71 | 0.30 (-0.04 to 0.64) | 83 | 0.12 (-0.18 to 0.41) | 0.41 |
|  | No weight loss | 88 | 0.07 (-0.27 to 0.41) | 79 | -0.12 (-0.45 to 0.21) | 0.43 |
| Waist circumference, cm | Weight loss | 71 | 0.83 (-0.43 to 2.09) | 82 | 1.69 (0.22 to 3.16) | 0.38 |
|  | No weight loss | 88 | 0.63 (-0.64 to 1.90) | 81 | 0.93 (-0.53 to 2.38) | 0.76 |
| Hip circumference, cm | Weight loss | 71 | 1.45 (0.56 to 2.35) | 82 | 1.99 (0.95 to 3.03) | 0.45 |
|  | No weight loss | 88 | 0.19 (-0.89 to 1.26) | 81 | 0.26 (-0.48 to 1.01) | 0.91 |
| Waist-to-hip ratio | Weight loss | 71 | -0.005 -(0.015 to 0.005) | 82 | -0.000 (-0.011 to 0.011) | 0.51 |
|  | No weight loss | 88 | 0.004 (-0.005 to 0.014) | 81 | 0.006 (-0.006 to -0.017) | 0.86 |
| Subcutaneous abdominal fat area, cm^2^ | Weight loss | 70 | 12.3 (3.8 to 20.8) | 83 | 14.0 (4.4 to 23.7) | 0.79 |
|  | No weight loss | 89 | -6.2 (-15.9 to 3.4) | 81 | -1.8 (-9.6 to 6.1) | 0.47 |
| Intra-abdominal fat area, cm^2^ | Weight loss | 70 | 9.3 (4.4 to 14.2) | 83 | 7.4 (3.0 to 11.8) | 0.57 |
|  | No weight loss | 89 | 4.3 (-0.6 to 9.1) | 81 | 7.8 (2.5 to 13.0) | 0.33 |
| Total abdominal fat area, cm^2^ | Weight loss | 70 | 21.6 (9.9 to 33.2) | 83 | 21.5 (9.0 to 33.9) | 0.99 |
|  | No weight loss | 89 | -2.0 (-15.1 to 11.1) | 81 | 6.0 (-4.6 to 16.6) | 0.35 |

^a^ Change in adiposity measure during the intervention from 0 to 12 months. “Weight loss” is defined as weight loss ≥3%; “No weight loss” is defined as weight gain ≥3% (increase) or absolute weight change <3% (maintenance).

^b^ Change in adiposity measure during follow-up, i.e., 24-month adiposity measure – 12-month adiposity measure

^c^ P value for the test of significance that the difference between the mean of MOD and HIGH group equals 0, by Student’s t-test

**Figure S6:** Additional average measurements over time for participants with complete data at each time point. Sample sizes based on data availability were: n=163 HIGH, n=159 MODERATE. Abbreviations: BMI, body mass index (kg/m^2^); WHR, waist-to-hip ratio.

**
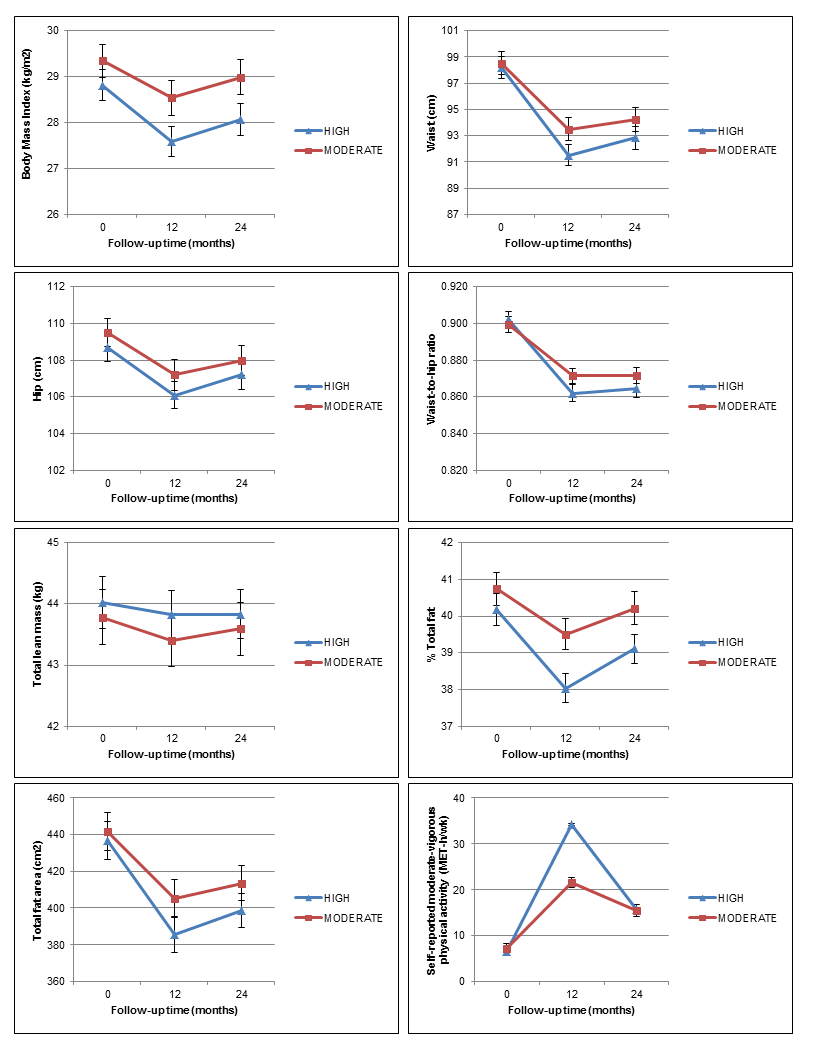
**

**
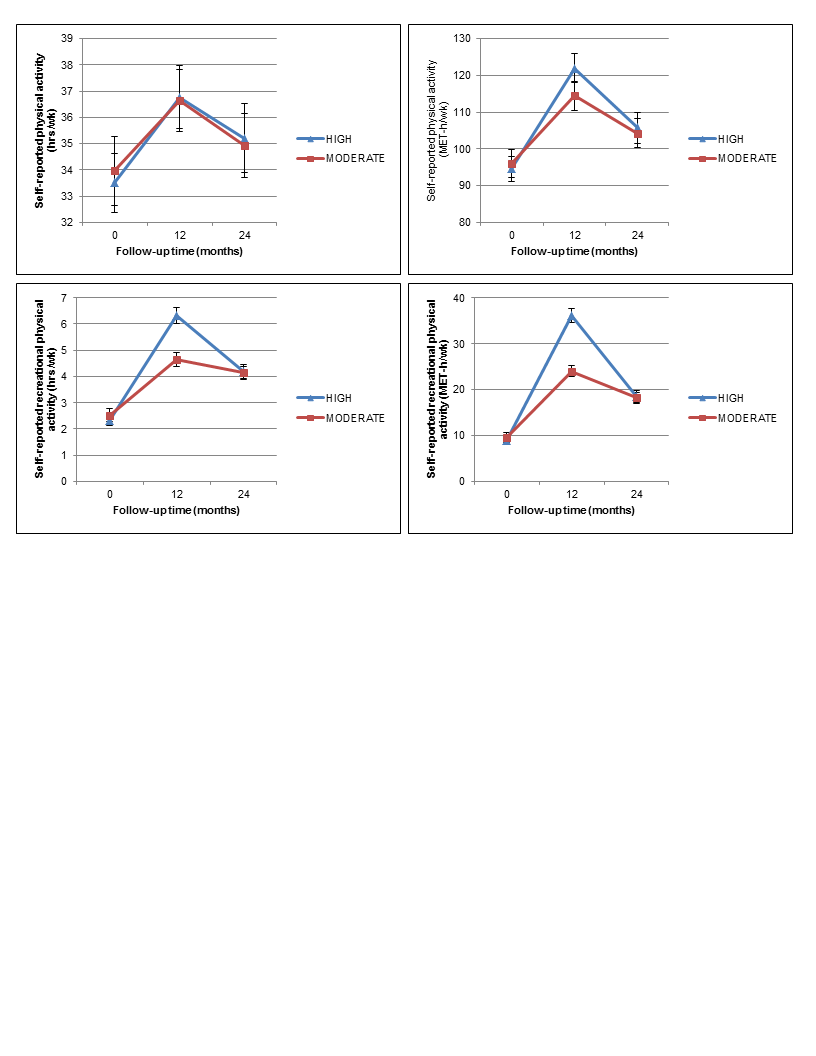
**
